# The GoodHope Ehlers Danlos Syndrome Clinic: development and implementation of the first interdisciplinary program for multi-system issues in connective tissue disorders at the Toronto General Hospital

**DOI:** 10.1186/s13023-021-01962-7

**Published:** 2021-08-10

**Authors:** Nimish Mittal, Daniel Santa Mina, Laura McGillis, Aliza Weinrib, P. Maxwell Slepian, Maxim Rachinsky, Stephanie Buryk-Iggers, Camille Laflamme, Laura Lopez-Hernandez, Laura Hussey, Joel Katz, Lianne McLean, Dmitry Rozenberg, Louis Liu, Yvonne Tse, Colleen Parker, Arnon Adler, George Charames, Robert Bleakney, Christian Veillette, Christopher J. Nielson, Sandra Tavares, Stephanie Varriano, Juan Guzman, Hanna Faghfoury, Hance Clarke

**Affiliations:** 1 GoodHope Ehlers Danlos Syndrome Program, Toronto General Hospital, 200 Elizabeth Street, Toronto, ON M5G 2C4 Canada; 2 Temerty Faculty of Medicine, Division of Physical Medicine and Rehabilitation, University of Toronto, Toronto, ON Canada; 3 Faculty of Kinesiology and Physical Education, University of Toronto, Toronto, ON Canada; 4 Department of Anaesthesiology and Pain Medicine, University Health Network, Toronto, ON Canada; 5 Department of Psychology, York University, Toronto, ON Canada; 6 Temerty Faculty of Medicine, Division of Respirology, Ajmera Transplant Program, Toronto General Hospital Research Institute, UHN, University of Toronto, Toronto, ON Canada; 7 Division to Temerty Faculty of Medicine, Division of Gastroenterology, University Health Network, University of Toronto, Toronto, ON Canada; 8 Division of Cardiology, Peter Munk Cardiac Centre, University Health Network, University of Toronto, Toronto, ON Canada; 9 Joint Department of Medical Imaging, University of Toronto, Toronto, ON Canada; 10 Department of Surgery, Division of Orthopedics, University of Toronto, Toronto, ON Canada; 11 Department of Medicine, McMaster University, Hamilton, ON Canada; 12 Temerty Faculty of Medicine, Division of Medical Genetics, University of Toronto, Toronto, ON Canada; 13 University of Toronto Centre for the Study of Pain, University of Toronto, Toronto, ON Canada

**Keywords:** Ehlers Danlos Syndrome, Hypermobility spectrum disorders, Connective tissue disorders, Diagnosis and treatment

## Abstract

Ehlers-Danlos Syndrome (EDS) are a heterogeneous group of genetic connective tissue disorders, and typically manifests as weak joints that subluxate/dislocate, stretchy and/or fragile skin, organ/systems dysfunction, and significant widespread pain. Historically, this syndrome has been poorly understood and often overlooked. As a result, people living with EDS had difficulty obtaining an accurate diagnosis and appropriate treatment, leading to untold personal suffering as well as ineffective health care utilization. The GoodHope EDS clinic addresses systemic gaps in the diagnosis and treatment of EDS. This paper describes a leap forward—from lack of awareness, diagnosis, and treatment—to expert care that is tailored to meet the specific needs of patients with EDS. The GoodHope EDS clinic consists of experts from various medical specialties who work together to provide comprehensive care that addresses the multi-systemic nature of the syndrome. In addition, EDS-specific self-management programs have been developed that draw on exercise science, rehabilitation, and health psychology to improve physical and psychosocial wellbeing and overall quality of life. Embedded into the program are research initiatives to shed light on the clinical presentation, underlying mechanisms of pathophysiology, and syndrome management. We also lead regular educational activities for community health care providers to increase awareness and competence in the interprofessional management of EDS beyond our doors and throughout the province and country.

## Background

Ehlers Danlos Syndromes (EDS) are a heterogeneous group of hereditary connective tissue disorders characterized by the abnormal formation and/or assembly of collagen, fibrillin and elastin in the body [1]. Defects in these connective tissue fibres can result in multi-systemic manifestations, ranging from loose, painful joints and abnormal stretchy, fragile skin to life-threatening complications [2–4]. Historical data estimates the combined prevalence in adults to be approximately 1 in 5000 [5], with subtype prevalence varying from 1 in 20,000 individuals in classical EDS [6] to 1 in 50,000 in vascular EDS [7]. Recent studies have raised the question of underestimation and have suggested a potentially higher prevalence in the general population [8]. Given the lack of awareness, understanding and expertise of health care providers in EDS, many cases are likely undiagnosed, resulting in probable under-reporting of this disorder, and its actual prevalence may be higher than reported in the current literature [8].

While some individuals born with EDS remain asymptomatic for their entire lives, many develop a myriad of symptoms, including generalized chronic pain [4], cognitive dysfunction, chronic fatigue and exhaustion [9], gastrointestinal symptoms [2], autoimmune dysfunction [10], orthostatic intolerance syndromes [11], respiratory symptoms and complications [12], abnormal degranulation of mast cells [13], and vestibular and proprioceptive disturbances [14]. These symptoms range in severity, from mildly impairing to severely disabling, leaving some people with EDS unable to work, attend school, or participate in social events [15]. Despite the severe impact of EDS, the nature and causes of multi-system dysfunction in EDS are not well understood; however, a small but growing body of research on EDS/HSD suggests the etiology and pathophysiology are complex and multifactorial.

### Challenges with diagnosis and treatment

Medical education on EDS has historically been minimal, thus there is a lack of knowledge and expertise to diagnose this condition. As clinicians have struggled to arrive at a diagnosis and find effective treatment options for people with EDS, research has shown that these patients feel invalidated and defeated, and often have a long history of engagement with the medical system [16]. It is common for patients to spend many years navigating the health care system in search of care, and reports estimate that the average time to diagnosis ranges from 4 to 16 years [17]. Moreover, there has been a history of health care professionals discounting patients’ symptoms and attributing them to psychological problems, as the different symptoms in multiple systems were often not united under a unifying diagnosis [16]. Despite several revisions to the EDS classification, many patients do not fit the current diagnostic categories in a clear-cut fashion and continue to struggle in their quest for a diagnosis [18]. Patients’ exhaustive search for care is associated with increased health services utilization and adverse health outcomes secondary to delayed diagnosis and intervention [19]. It is within this context that the need for the GoodHope Ehlers Danlos Syndrome Clinic arose.

Rare complex medical disorders like EDS have always struggled with inadequate health care provisions. Multisystemic needs of persons living with EDS require care from multiple practitioners and poses a challenge of care coordination. Further, the typical treatment approaches designed to treat common conditions may not be relevant to individuals with EDS as they have structurally defective connective tissue that forms building blocks of nearly every organ system in the body. Globally, the care of individuals with EDS has been fragmented without a centralized clinic, and there is a lack of specialized interdisciplinary programs to address this health care and knowledge gap in EDS. The GoodHope EDS clinic at the Toronto General Hospital is the first unique one stop interdisciplinary model of health care delivery that could serve as a template to other Canadian provinces and interested cities internationally looking to improve the care for this complex population.

### Establishment of the GoodHope EDS clinic

Advocacy efforts by people living with EDS in Ontario, Canada, led to the creation of the Ehlers-Danlos Syndrome Expert Panel at the request of the provincial Ministry of Health and Long-Term Care (MOHLTC) in July of 2015. People in Ontario living with EDS gave voice to their complex healthcare needs and identified the lack of specialized care in Canada[20]. The panel expressed their concern with the lack of knowledge of EDS among health care providers, the lack of sufficient practitioners with expertise in the management of all EDS variants, and challenges related to health care continuity and interdisciplinary collaboration in practice.

Based on the expert panel report, the MOHLTC announced provincial funding to open one adult and one pediatric EDS clinic in Toronto as part of the “Patients First: Action Plan for Health Care” [21]. The adult EDS clinic opened in 2017 under the the Rare Diseases umbrella at the Toronto General Hospital, part of the University Health Network. In addition to provincial support, the GoodHope EDS clinic received philanthropic support to develop a pioneering program of EDS research.

**Fig. 1:**
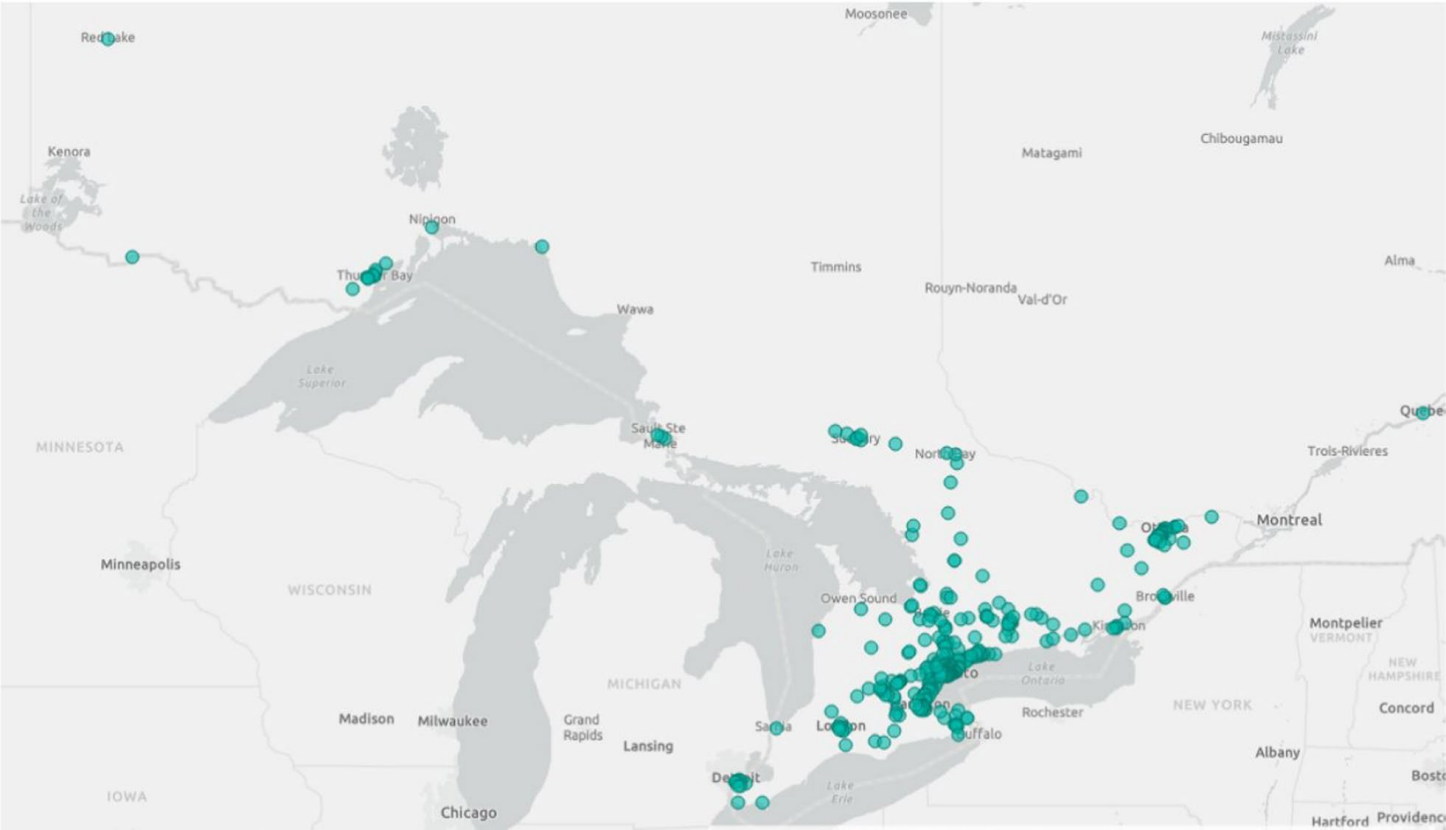
The geographic area of Ontario highlighting areas of residence of patients that were seen at the GoodHope EDS Clinic

The catchment area for treatment eligibility in the GoodHope EDS Clinic encompasses the entire geographic area of Ontario—a Canadian province of over 14 million people in a geographic area larger than France (Fig. 1). To date, more than 1,000 patients have been assessed for possible EDS/HSD. The EDS clinic continues to follow approximately 400 patients that were confirmed to meet the criteria for a diagnosis of EDS or Generalized Hypermobility Spectrum Disorders (G-HSD). For these patients, key goals include the timely delivery of expert, specialized, multi-systemic, and biopsychosocial health care**.**

### The GoodHope EDS team

Given the complex nature of EDS diagnosis and treatment, the EDS clinic team is comprehensive:

- The *core assessment and follow up team* consists of physicians, fellows, and nurse practitioners. This group is tasked with: [1] conducting intake assessments to determine diagnosis and eligibility for treatment at our program; and [2] providing a central hub for follow up visits as needed.
- The team of *medical specialists* includes physicians specialized in physical medicine and rehabilitation, anesthesia and pain management, gastroenterology, cardiology, respirology, immunology, genetics, neuroradiology, spine surgery, and vascular surgery.
- A *genetic counsellor* provides genetic testing and related counselling.
- A team of *physiotherapists and kinesiologists* provide EDS-specific exercise and rehabilitation programming to improve joint stability and strength and help patients reach functional goals.
- *Clinical psychologists deliver* programs to support patients’ mental health.
- A *social worker* connects people living with EDS with community supports and resources.
- A *dietitian* provides specialized food plans to improve nutrition and digestion.

In addition to this complement of clinical and administrative staff, a *patient and family advisory group* provide the patient perspective to hospital administrators and EDS clinicians to ensure that care is oriented to the evolving needs of this population and sets goals for program development. The advisory council meets regularly so that dialogue can occur between all stakeholders.

### Initiating care: patient referral and initial assessment

**Fig. 2:**
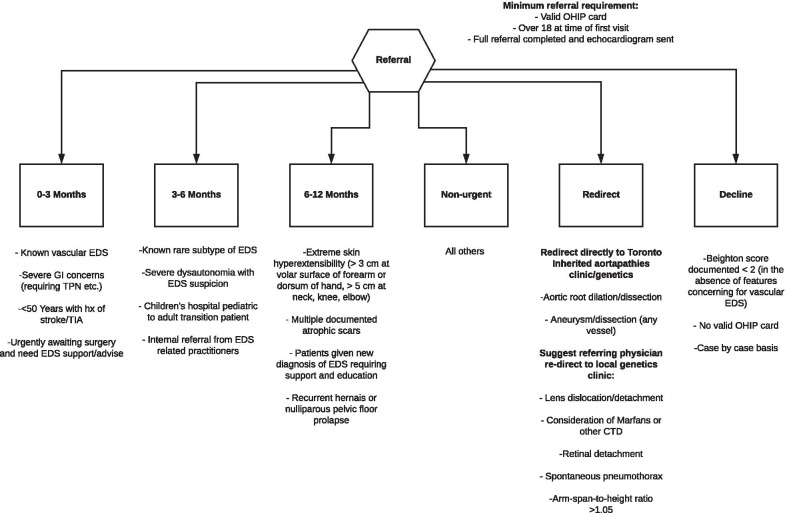
The triaging process of a referral to the GoodHope EDS Clinic

**Fig. 3:**
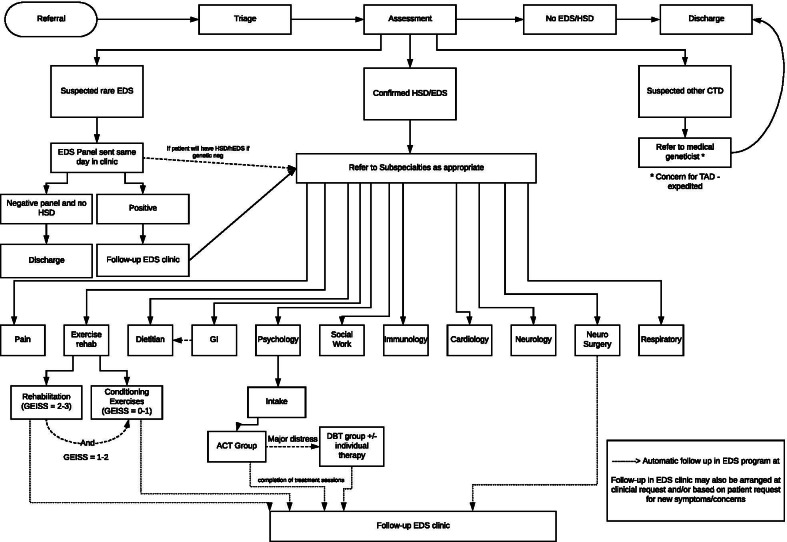
A detailed diagram of the interdisciplinary patient service flow

Referrals to the GoodHope EDS clinic from primary care physicians are reviewed and triaged into categories of booking priority based on urgency (Fig. 2). Patients are booked for a comprehensive assessment that includes Beighton score testing, which is conducted based on standard landmarks using an electronic goniometer, as per the guidelines of the International Consortium of EDS. Patients are then tested for hypermobility of other common large and small joints not included in the Beighton score [22]. Additionally, systematic manifestations of connective tissue abnormality are documented in detail. If the patient meets the clinical criteria for EDS, molecular testing is scheduled to confirm EDS subtypes, such as COL3A1, COL5A1, and COL5A2. For the patients who meet the 2017 clinical criteria for any subtype of EDS including hypermobile EDS (hEDS) or have a clinical phenotype consistent with generalized hypermobility spectrum disorders (G-HSD), the clinic formulates individualized care plan that involves the breadth of medical and clinical specialists in the EDS clinic. Currently, there is insufficient evidence to determine if G-HSD and hEDS are part of the same hereditary connective tissue disorder or not, and the GoodHope EDS clinic follows all patients that meet the 2017 clinical criteria for G-HSD. For those patients with hypermobility but with clinical features suggestive of another genetic syndrome (e.g., osteogenesis imperfecta), consultation with a geneticist is initiated. This single, comprehensive clinic approach facilitates early diagnostic confirmation and timely access to multisystemic interprofessional care for individuals living with EDS. A detailed diagram of the interdisciplinary patient service flow is shown in Fig. 3.

### Individualized care plan

Patients with complex gastrointestinal, respiratory, cardiac, urological, chronic pain, respiratory symptoms, and/or widespread generalized allergic symptoms are provided with evidence-based recommendations and referred to dedicated sub-specialty within the EDS clinic. Patients with transient neurological symptoms and headaches, if deemed appropriate, are referred for a battery of radiological imaging and neurophysiological testing for the identification of possible craniocervical junction abnormalities. In the case of demonstrable abnormalities, patients are referred to spine services for consultation and management. Specific pathways of care for common EDS sequela are described hereafter.

#### EDS orthostatic intolerance assessment pathway

Previous studies have shown that chronic orthostatic intolerance syndromes are a prevalent clinical finding in patients with EDS, including vasovagal syncope, postural orthostatic tachycardia syndrome (POTS) and orthostatic hypotension (OH)/autonomic failure [23]. The EDS GoodHope clinic has established collaborative partnerships with other subspecialized health care providers (JG) and other institutions to provide additional diagnosis and management support for these conditions. Orthostatic vitals are captured during the initial clinical assessment in the GoodHope EDS clinic and detailed screening is performed to identify patients with structural heart disease, arrhythmias and chronic orthostatic intolerance syndromes and dysautonomia. For patients that screen positive, a referral to the Syncope and Autonomic Disorders Clinic affiliated to the Arrhythmia Services at Hamilton Health Sciences/McMaster University (Hamilton, ON, Canada) and/or the Autonomic Clinic at Women’s College Hospital (Toronto, ON, Canada) is made for more detailed cardiovascular assessment. If the clinical diagnosis is confirmed, the providers determine the need for further testing and develop an individualized management plan. Follow up is provided in person or using Telemedicine Services, including Home Video Visits, an e-consult platform from the Ontario Telemedicine Network (OTN).

#### EDS gastrointestinal pathway

Gastrointestinal symptoms such as abdominal pain, nausea, vomiting, bloating and altered bowel habits are common in patients with EDS. Specifically, disorders of gut-brain interaction, such as irritable bowel syndrome, appear to be more common in this patient population and can often be challenging to manage [2]. The gastroenterology (GI) service as part of the GoodHope EDS clinic is dedicated to the diagnosis and management of these disorders. There are three gastroenterologists who have expertise in neurogastroenterology and the management of these conditions. The physicians assess all new consults in the clinic and determine their need for diagnostic testing. These patients often undergo a variety of gastrointestinal investigations, which may include specialized motility testing such as esophageal manometry, ambulatory esophageal pH testing, gastric emptying studies and anorectal manometry. Based on the results, both pharmacologic and non-pharmacologic approaches are then used in the management of their symptoms. Often other services available in the GoodHope EDS Clinic are then asked to precisely manage their gastrointestinal symptoms including dietitians and psychologists.

#### EDS pain pathway

Chronic multi-site pain is one of the most frequent and debilitating symptoms for people with EDS. The pathogenesis of pain in EDS is multifactorial and not fully understood [4]. Persons living with EDS suffer not only from nociceptive pain secondary to tissue sprains and injuries from joint laxity, but also commonly report significant bothersome neuropathic pain and hyperalgesia [4]. Recent studies have reported small fibre neuropathy as a standard feature that may explain widespread neuropathic pains to some extent [24]. The EDS pain service has two pain physicians (HC and MR) with expertise in the diagnosis and management of complex chronic pain disorders. The patients undergo a comprehensive pain focused assessment and are provided with an inter-disciplinary approach to pain management, combining physician and allied health team members' input with focus on the quality of life and goal setting both in the short and long term.

#### EDS respirology pathway

Respiratory symptoms in EDS can be non-specific, including dyspnea, cough, exercise intolerance, voice hoarseness, wheezing and chest tightness [12]. Respiratory disorders described in EDS include asthma and atopic symptoms, in part, from mast cell dysregulation [13] laryngeal involvement [25], respiratory muscle weakness, sleep apnea [26], pneumonia and bullous lung disease [12]. People living with EDS are often assessed by respirology in the community with routine investigations often performed, including pulmonary function testing and radiological imaging. Additional investigations as part of the work-up by respirology service of the GoodHope EDS clinic may include muscle strength testing, ultrasound visualization of the diaphragm, direct visualization of the vocal cords and airways (laryngoscopy or bronchoscopy), sleep study, and dedicated inspiratory and expiratory computed tomography to assess for any significant airway collapse [26]. An emphasis of clinical service and future research within this pathway includes the examination of respiratory muscle training in collaboration with physiotherapy, given emerging evidence of benefit in those with significant reductions in respiratory muscle strength [26].

#### EDS exercise and rehabilitation pathway

One of the significant challenges that people with EDS face is remaining active and robust, despite the fear of recurrent injury. Exercise and rehabilitation are indicated for people with EDS to help manage the movement dysfunction, chronic pain, and deconditioning that result from diffuse joint instability, recurrent subluxations, and kinesiophobia [14]. The overarching mission of the GoodHope Exercise and Rehabilitation (GEAR) service of the GoodHope EDS clinic is to improve the physical function and psychosocial well-being of people with EDS through structured exercise. There has been very little empirical data to support or refute the value or direct the delivery of such services for people with EDS [27]. The GEAR service is developing and testing new exercise and rehabilitation programs for this population. For entry into the appropriate stream of the GEAR program, patients’ symptom burden and overall functioning are assessed using the GoodHope EDS Impairment and Interference Scale (GEIIS; Table 1). Rehabilitation and conditioning-based exercises are tailored to patients’ needs. For example, patients who are more restricted in their functioning may start with the physical rehabilitation, whereas patients who are higher functioning engage in general conditioning and fitness programs (Tables 2). Given the variability of dysfunction and symptoms for a person with EDS, elements of both rehabilitation and general conditioning may be indicated.

**Table 1:** GoodHope EDS Impairment and Interference Scale (GEISS)

Score	Description
0	Able to participate in all self-care, occupational, and leisure activities and exercise without restriction
1	Able to participate in self-care activities as well as activities requiring light to moderate exertion (e.g., light housework, office work, or gentle exercise); unable to perform, or difficulty with, physically strenuous activities
2	Able to participate in self-care activities but unable to perform, or difficulty with, occupational and leisure activities of mild or moderate exertion
3	Unable to perform some self-care activities and/or requires assistance with others; limited activities beyond the bed or chair most of the time
4	Unable to perform all self-care activities; not ambulatory

**Table 2:** GEAR program streams

Program Stream	GEIIS Score	Clinician
Exercise	0–1	Kinesiologist or Physiotherapist
Rehabilitation	2–3	Physiotherapist
Combined (Exercise & Rehabilitation)	1–2 with requirement for conditioning ***and*** impairment-based exercises	Physiotherapist ± Kinesiologist
One-time active lifestyle counselling session	0–3 ***and*** unable to attend in-hospital Exercise and/or Rehabilitation sessions	Physiotherapist or Kinesiologist

Note: the program is overseen by a physical medicine and rehabilitation physician who provides initial physical assessment of the patient, including contraindications and goals of treatment, and is available for consult regarding the exercise and rehabilitation care

Rehabilitation care focuses on assessing neuromotor and functional performance specific to a patient's goals and problematic joints with self-management strategies to address emerging pain in affected joints. General conditioning exercises are prescribed to complete independently (e.g., at home) and aim to improve overall strength, endurance and function. Resistance exercises focus on utilizing major muscle groups and multi-joint movements to improve strength and stability, whereas aerobic exercise prescriptions target moderate-intensity physical activity for 150 min per week. Modification of exercises is routinely incorporated to address patients' risk of orthostatic intolerance, gastrointestinal symptoms, multiple joint symptom involvement, respiratory symptoms, and pain.

#### EDS psychological service pathway

People living with EDS have high rates of depression and anxiety [28], similar to people living with other chronic illnesses [29]. The high prevalence of emotional distress in people with EDS/HSD may be primarily due to the stress of living with chronic illness or maybe due to shared physiological pathways that lead to both physical and emotional symptoms (just as inflammation is a shared vulnerability that links heart disease and depression). People living with EDS have often had negative experiences with mental health providers, such as the attribution of poorly understood symptoms and associated distress to somatization. The EDS psychology service uses a biopsychosocial framework to compassionately and effectively support patients in coping with the significant physical, emotional, and social challenges of living with EDS. The EDS psychological services are informed by patient feedback so that care is patient centered.

The psychological treatments at the GoodHope EDS clinic emphasize mindfulness and acceptance-based approaches. The psychology service has developed an Acceptance and Commitment Therapy (ACT) workshop that is specifically tailored for people with EDS [30]. One-day ACT workshops have been shown to reduce distress while improving engagement with meaningful activities and health behaviour in people living with chronic health conditions [30]. In addition, the psychology service offers a brief Dialectical Behavior Therapy (DBT) informed skills group for patients who are interested in repeatedly meeting to practice coping skills to reduce emotional distress. DBT was initially developed to treat emotional dysregulation but is being used with higher frequency in pain management and behavioural medicine. DBT skills such as self-validation, radical acceptance, and distress tolerance have been well received by people living with EDS. Group programs give patients the valuable opportunity to discuss their unusual and sometimes isolating life experiences in a community of “EDSers” who understand, all in the context of a supportive and non-judgmental therapeutic environment.

### Clinic development lessons learned

The GoodHope EDS clinic represents a unique interdisciplinary patient centred stepped care model of evidence-based service delivery ultimately made feasible by funding from the MOHLTC and philanthropic support as many persons living with EDS are not able to routinely afford these interdisciplinary multimodal services. Owing to the complex multisystemic needs of persons living with EDS, tertiary hospital centres or medical centres with a clear process of access to multiple specialities are at an advantage to provide required care and support. In the early days of establishment, GoodHope EDS clinic faced several operational challenges related to medical specialist engagement and interdisciplinary collaborations as vast majority of medical providers do not have the necessary knowledge or skill set in this specialized area of medicine. Recently, Covid-19 related changes have necessitated virtual delivery of self-management services and provided as an opportunity to create virtually delivered self-management modules to serve larger catchment areas in the long term.

### Education, research initiatives, and future directions

Knowledge dissemination to health care providers at the community level is a crucial objective as currently, community healthcare providers are not able to support the ongoing care and management of rare complex disorders. The GoodHope EDS clinic organizes regular seminars and lectures at local and provincial levels every three months to disseminate best clinical practices in EDS. Examples of seminar and lecture topics include mast cell disorders, histopathological diagnosis of connective tissue disorder and POTS in EDS. Partnerships with the Toronto Academic Pain Institute (TAPMI) chronic pain hub, are being built in order to create patient facing on-line educational content.

The comprehensive and ongoing care provided by the GoodHope EDS clinic provides a unique opportunity and capability to conduct prospective, longitudinal research, which is ideal for understanding the evolution of symptoms and complications; estimating risk; tracking patient outcomes, predicting prognosis; and evaluating treatment/program efficacy/effectiveness. A comprehensive clinical-research database (REB No: 18–5850)has been established to serially collect data at each patient interaction to actively track patients’ changes in health and contribute to a stronger/better understanding of the natural history of EDS within the GoodHope EDS clinic framework. Planned analyses include those that will advance our understanding of EDS. Specifically, we aim to use the clinical-research database to better characterize homogenous anatomic variables to stratify hEDS and distinguish it from HSD, identify constructs of physical and functional impairment in different subtypes of EDS, and risk factors that predict severity of the disease. Moreover, the GoodHope EDS Clinic has a team of basic science researchers and geneticists to investigate causative pathophysiological mechanisms underlying EDS, identify novel genes, genetic variabilities or other molecular markers, and develop innovative treatments tailored to individuals with compromised structural integrity of connective tissues.

In addition to the ongoing and planned research, future directions of GoodHope EDS clinic include expansion of digital health services to increase accessibility to timely and personalized care. This innovative approach currently provides access to virtual psychological services, via a third-party digital mobile health platform [31, 32], to those with the greatest need, identified real-time with a virtual screening tool using a stepped care model. The clinic plans to expand virtual health care service delivery for all self-management programs. Optimization of digital healthcare support will be informed by feasibility and usability data, as well as their success with respect to improving outcomes of self-management programs.

## Conclusions

The diagnosis of EDS continues to be a road that is challenging to navigate for patients. The goal of Goodhope EDS clinic is to transform the diagnosis and management of EDS from one that is fraught with misunderstanding and confusion to a process that is refined and scientifically grounded. The GoodHope EDS clinic is based on the principle that a comprehensive, humanistic, interdisciplinary, evidence-based approach that empowers psychological and functional health is the path forward to helping the entire spectrum of people living with EDS.

## Data Availability

Not applicable.

## Abbreviations

ACT: Acceptance and Commitment Therapy
DBT: Dialectical Behaviour Therapy
EDS: Ehlers Danlos Syndrome
GEAR: GoodHope Exercise And Rehabilitation
GEIIS: GoodHope EDS Impairment and Interference Scale
GI: Gastroenterology
G-HSD: Generalized Hypermobility Spectrum Disorders
MOHLTC: Ministry of Health and Long-Term Care
OH: Orthostatic Hypotension
OTN: Ontario Telemedicine Network
POTS: Postural Orthostatic Tachycardia Syndrome
